# Actionable Gene Alterations Identified in Patients With Malignant Melanoma by Targeted Sequencing in Japan

**DOI:** 10.1200/PO-24-00437

**Published:** 2025-01-17

**Authors:** Takuro Noguchi, Shin Ariga, Rika Moku, Junko Kikuchi, Toraji Amano, Takuya Maeda, Kosuke Ishikawa, Taku Maeda, Akihiko Shiiya, Tomohiro Goda, Yoshihito Ohhara, Kanako Hagio, Yusuke Saito, Kanako C. Hatanaka, Yutaka Hatanaka, Jun Taguchi, Satoshi Takeuchi, Yasushi Shimizu, Ichiro Kinoshita

**Affiliations:** ^1^ Department of Medical Oncology, Faculty of Medicine and Graduate School of Medicine, Hokkaido University, Sapporo, Hokkaido, Japan; ^2^ Department of Medical Oncology, Hokkaido University Hospital, Sapporo, Hokkaido, Japan; ^3^ Division of Clinical Cancer Genomics, Hokkaido University Hospital, Sapporo, Hokkaido, Japan; ^4^ Department of Dermatology, Faculty of Medicine and Graduate School of Medicine, Hokkaido University, Sapporo, Japan; ^5^ Department of Plastic and Reconstructive Surgery, Faculty of Medicine and Graduate School of Medicine, Hokkaido University, Sapporo, Japan; ^6^ Center for Development of Advanced Diagnostics, Hokkaido University Hospital, Sapporo, Japan

## Abstract

**PURPOSE:**

Precision medicine plays an important role in the treatment of patients with advanced melanoma. Despite its high incidence in White patients, advanced melanoma is rare in Asian countries, hampering prospective clinical trials targeting the Asian population. This retrospective study aimed to elucidate the real-world molecular diagnoses and outcomes of Japanese patients with melanoma using comprehensive genome profiling (CGP).

**MATERIALS AND METHODS:**

Patients with melanoma who completed standard anticancer medical treatments (including those expected to complete the treatments) underwent CGP, which is covered by the National Health Insurance. We analyzed the results and clinical annotations of 569 patients registered before August 2023 in a national database.

**RESULTS:**

Skin, mucosal, and uveal melanomas accounted for 64%, 28%, and 7% of cases, respectively. Patients with *BRAF*, *NRAS*, *NF1*, and *KIT* variants represented 25%, 20%, 17%, and 17%, respectively. Eighty-two percent of *BRAF*, 97% of *NRAS*, 69% of *NF1*, and 54% of *KIT* were actionable alterations (ie, *BRAF* classes I, II, and III, *NRAS* Q61, G12, G13, *NF1* loss-of-function, *KIT* gain-of-function variants). *BRAF* V600E/K variants occurred in 22% of skin and 2% of mucosal melanomas, but not in uveal melanomas. The mean tumor mutation burden in cutaneous melanomas was 4.2 variants/Mb. Patients previously treated with BRAF-targeted therapy harbored amplifications of *BRAF* and cell cycle genes more frequently than therapy-naive patients. Thirty-six patients (6.3%) were treated following the molecular tumor board (MTB) recommendations.

**CONCLUSION:**

Actionable gene alterations in *BRAF*, *NRAS*, *NF1*, and *KIT* are common in Japanese patients with melanoma. However, few patients were treated according to the MTB recommendations, suggesting that there is an unmet need to increase accessibility to gene-matched clinical trials in Japan.

## INTRODUCTION

The efficacy of anticancer therapies for advanced melanomas has been limited in the past, with an expected survival for patients on therapy of less than a year.^1^ However, the advent of immune checkpoint inhibitors (ICIs) and BRAF/MEK inhibitors has dramatically changed treatment outcomes.^2-4^ Currently, approximately 50% of patients with unresectable or metastatic cutaneous melanomas survive for more than 5 years.^2^ Genomic biomarkers that stratify responses to therapies have been extensively explored. A high tumor mutation burden (TMB) is associated with the efficacy of ICIs that target neoantigens derived from somatic variants.^5^ Malignant melanoma is characterized by a high TMB resulting from the accumulation of sun exposure–derived DNA damage, which, in turn, endows it with sensitivity to ICIs.^6^ Alternatively, the *BRAF* V600E and V600K variants are additional actionable genomic biomarkers that predict responses to BRAF/MEK inhibitors. Although 40%-50% of melanomas in Western countries harbor these variants,^7,8^ the incidence in Asian countries is reportedly lower at 30%-40%.^9,10^

**CONTEXT**

**Key Objective**
Does health insurance–covering comprehensive genome profiling (CGP) benefit patients with advanced melanoma in Japan?
**Knowledge Generated**
Although the molecular tumor boards offered therapy recommendations for one third of the patients, only 36 of 569 patients (6%) were treated with the recommended drugs. Detailed therapy information was available for 29 of the 36 patients, and 10 received therapies other than the US Food and Drug Administration–approved medications.
**Relevance**
Currently, CGP plays a limited role in the treatment of patients with advanced melanoma in Japan. However, our study revealed that most patients harbored other actionable variants, such as *NRAS*, *KIT*, and *NF1*. Thus, easy access to targeted drugs, either under authoritative approval or in clinical trials, is an unmet need in the treatment of melanoma in Japan.

Comprehensive genome profiling (CGP) has revealed common oncogenic variants in *BRAF*, *NRAS*, and *NF1* in cutaneous melanomas, with a mean TMB of 17 variants/Mb and a ultraviolet radiation signature.^11^ This study of cutaneous melanomas also identified co-occurring or mutually exclusive gene alterations accompanied by these driver variants, suggesting the existence of complex regulatory networks in melanoma oncogenesis. However, in acral and mucosal melanomas, variants in *KIT* are more common (10%-30% of cases).^12-14^ By contrast, almost all uveal melanomas harbor gene alterations in either *GNAQ* or *GNA11* as driver genes that are absent in other melanomas.^15,16^

Despite well-characterized clinical and molecular information from Western countries, whether these data can be recapitulated in Asian populations remains poorly understood. In fact, malignant melanoma is a rare cancer in Japan, with a cutaneous melanoma incidence rate of 1.75 of 100,000 person-years.^17^ Immunotherapy efficacy is reportedly inferior in Asians compared with White patients. Several studies have suggested that the predominance of acral and mucosal melanomas in Asians may affect outcomes because they harbor a lower TMB.^18-20^ Although BRAF-targeted therapy has similar efficacy in Asians and White patients, the overall incidence of *BRAF* V600E/K variants is lower in Asians, which may hamper their opportunities for targeted therapy.

To address these concerns, we used the Center for Cancer Genomics and Advanced Therapeutics (C-CAT)'s national genomic database, which stores CGP results and relevant clinical information.^21^ Patients with advanced solid cancers are eligible for CGP covered by the National Health Insurance when they have nearly completed efficacious standard anticancer therapies and require genomic biomarkers for tumor-agonistic targeted therapies or are entering clinical trials.

In this study, we analyzed the clinical information and gene alterations in 569 Japanese patients with melanoma who underwent targeted clinical sequencing, including the recommendations provided by the molecular tumor board (MTB).

## MATERIALS AND METHODS

### Data Access

In Japan, CGP results paid for by the National Health Insurance are stored and managed by the C-CAT at the National Cancer Center, Japan,^21^ with written informed consent from the patients. At the data access date (August 2023), the approved CGP methods were FoundationOne CDx, FoundationOne Liquid CDx (F1Liquid) by Roche/Chugai, and NCC OncoPanel by Sysmex/National Cancer Center, Japan. Documents were saved at the medical institutes that performed CGP. After the approval of the research protocol by C-CAT (protocol number: CDU2023-016N) and the Internal Review Board at Hokkaido University Hospital (protocol number: 022-0380), we accessed the repository data in August 2023. Using the OncoTree classification system, we filtered the data by melanoma (MEL) for skin melanomas; by head and neck mucosal melanoma (HNMUCM), mucosal melanoma of the vulva/vagina (VMM), anorectal mucosal melanoma (ARMM), mucosal melanoma of the esophagus (ESMM), and mucosal melanoma of the urethra (URMM) for mucosal melanomas; and by uveal melanoma (UM), primary CNS melanoma (PCNSM), conjunctival melanoma (CM), and ocular melanoma (OM) for ocular melanomas, resulting in 571 cases. Skin MEL was further classified into acral melanoma (ACRM), congenital nevus (SKCN), cutaneous melanoma (SKCM), desmoplastic melanoma (DESM), lentigo maligna melanoma (SKLMM), melanoma of unknown primary (MUP), and spitzoid melanoma (SPZM). Of these, we excluded two SKCN cases. Accordingly, 569 melanoma cases were analyzed in this study. To compare melanomas in Japan and Western countries, we used data sets available at cBioPortal which predominately contains data from Western countries.^22^

### Data Processing

The original gene alteration data of Japanese melanomas were converted to the Mutation Annotation Format and analyzed using maftools^23^ in the R software. We investigated the clinical information of 569 cases and gene alterations in 562 cases, excluding those with CM and OM. The results from cBioPortal were filtered using 324 genes detectable by FoundationOne CDx and analyzed similarly.

We preprocessed the data according to the methods shown in Appendix Figure A4 to analyze the anticancer therapies. In the original data, treatment sequences were recorded by tagging as neoadjuvant, adjuvant, or the number of lines (first, second, etc). However, no neoadjuvant therapy with curative intent was approved during the registration period. Adjuvant therapies were continued for more than 1 year in some cases. We excluded these patients from subsequent clinical information analysis because we could not distinguish whether they received neoadjuvant or adjuvant therapies for curative or palliative purposes. To maintain data accuracy, we excluded cases with missing values for the required information. In the original data set, some patients were treated with a combination of first-line nivolumab and ipilimumab followed by second-line nivolumab. When subsequent nivolumab therapy was started 21-28 days after the last nivolumab and ipilimumab administration, it was regarded as maintenance and part of the previous line.

### Statistics

Gene variant frequencies between the two groups were analyzed using a Fisher's exact test. *P* values were adjusted using the Benjamini-Hochberg method. The frequency of patients who received anticancer therapies was analyzed using a Fisher's exact test. Statistical significance was set at *P* < .05.

## RESULTS

### The Study Cohort

**FIG A1.**
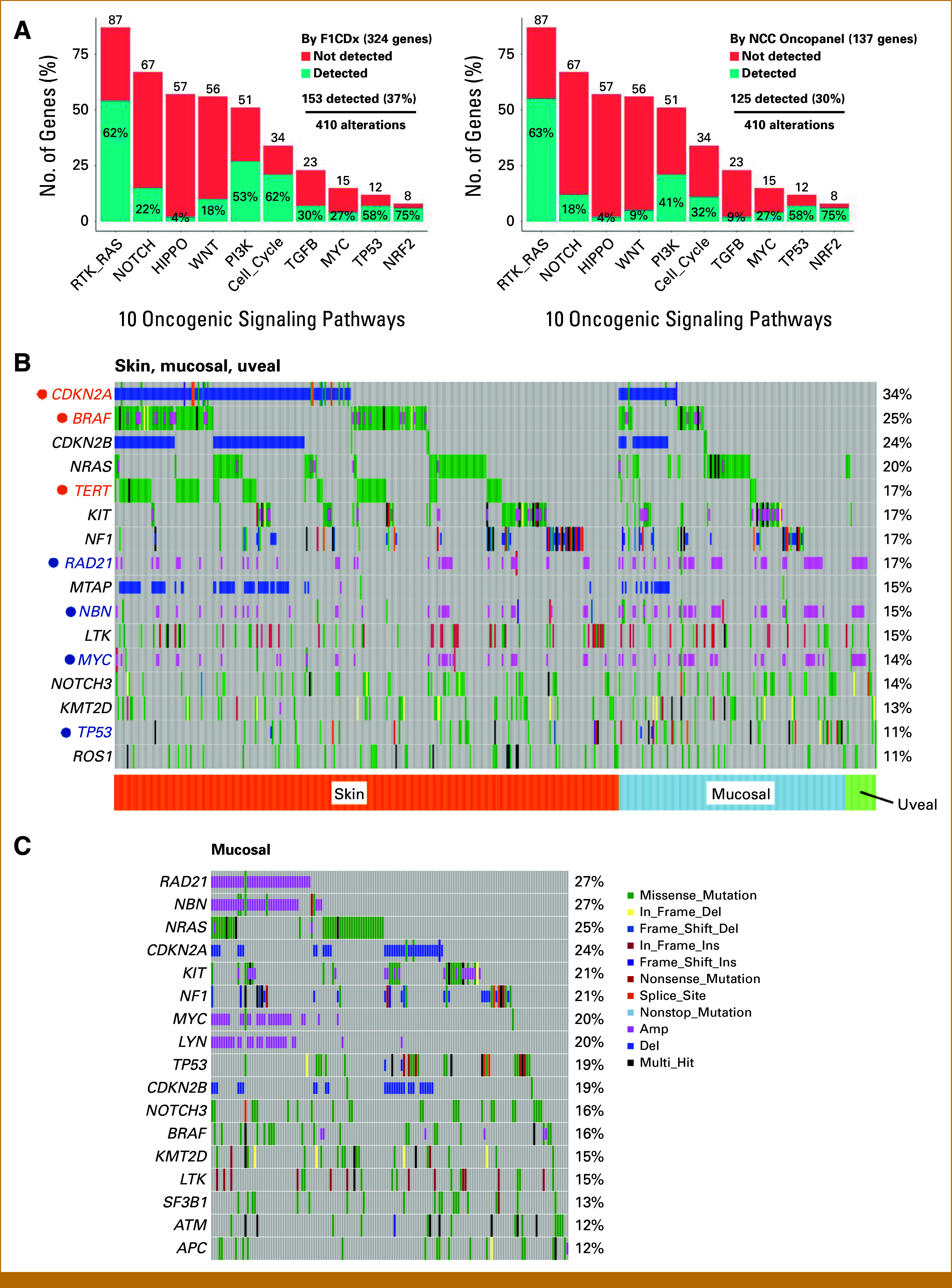
CGP test performance and results for Japanese melanomas. (A) Detectable gene alterations in 10 oncogenic pathways by FoundationOne CDx (F1CDx) and NCC OncoPanel are shown. Numbers above the bars represent genes involved in the pathways. (B) Most frequently altered genes in all Japanese melanomas. (C) Most frequently altered genes in Japanese mucosal melanomas.

Between October 2020 and May 2023, C-CAT registered CGP results from 569 patients with malignant melanoma (Table 1). The majority of samples were tissues from either primary or metastatic sites; however, 6% of the CGP results were from blood samples. During this period, the Japanese regulatory agency approved three targeted clinical sequencing panels: FoundationOne CDx, FoundationOne Liquid CDx (F1Liquid), and NCC OncoPanel, which analyzed 324, 324, and 137 genes, respectively. Although the NCC OncoPanel targets fewer genes than the FoundationOne panels, it identifies germline variants using blood as a reference and its performance in detecting variants in key oncogenic pathways is comparably sensitive (Appendix Fig A1A). The cohort consisted of approximately 64% skin melanoma, 28% mucosal melanoma, and 7% UM, including PCNSM. Because of the mid-study application of second-level OncoTree annotations, some skin melanoma cases were labeled only MEL.

**TABLE 1.** Study Patient Characteristics

Characteristic	Patients (N= 569)
Male/female, No.	291/278
Age, years, median (range)	66 (2-99)
Registration	October 2020-May 2023
Sample collection site, No. (%)	
Primary	269 (47)
Metastasis	263 (46)
Blood	34 (6.0)
Unknown	3 (0.5)
NGS panel, No. (%)	
FoundationOne CDx	480 (84)
NCC OncoPanel	55 (9.7)
F1Liquid CDx	34 (6.0)
Melanoma type, No.	
Skin	364
MEL	189
SKCM	99
ACRM	39
MUP	29
SKLMM	4
DESM	3
SPZM	1
Mucosal	159
HNMUCM	78
VMM	34
ARMM	25
ESMM	21
URMM	1
Uveal, CNS, ocular	46
UM	31
PCNSM	8
CM	5
OM	2

Abbreviations: ACRM, acral melanoma; ARMM, anorectal mucosal melanoma; CM, conjunctival melanoma; DESM, desmoplastic melanoma; ESMM, mucosal melanoma of the esophagus; HNMUCM, head and neck mucosal melanoma; MEL, melanoma; MUP, melanoma of unknown primary; NGS, next-generation sequencing; OM, ocular melanoma; PCNSM, primary CNS melanoma; SKCM, skin cutaneous melanoma; SKLMM, lentigo maligna melanoma; SPZM, spitzoid melanoma; UM, uveal melanoma; URMM, mucosal melanoma of the urethra; VMM, mucosal melanoma of the vulva/vagina.

### CGP Results From Japanese Malignant Melanomas by Subtypes

**FIG 1.**
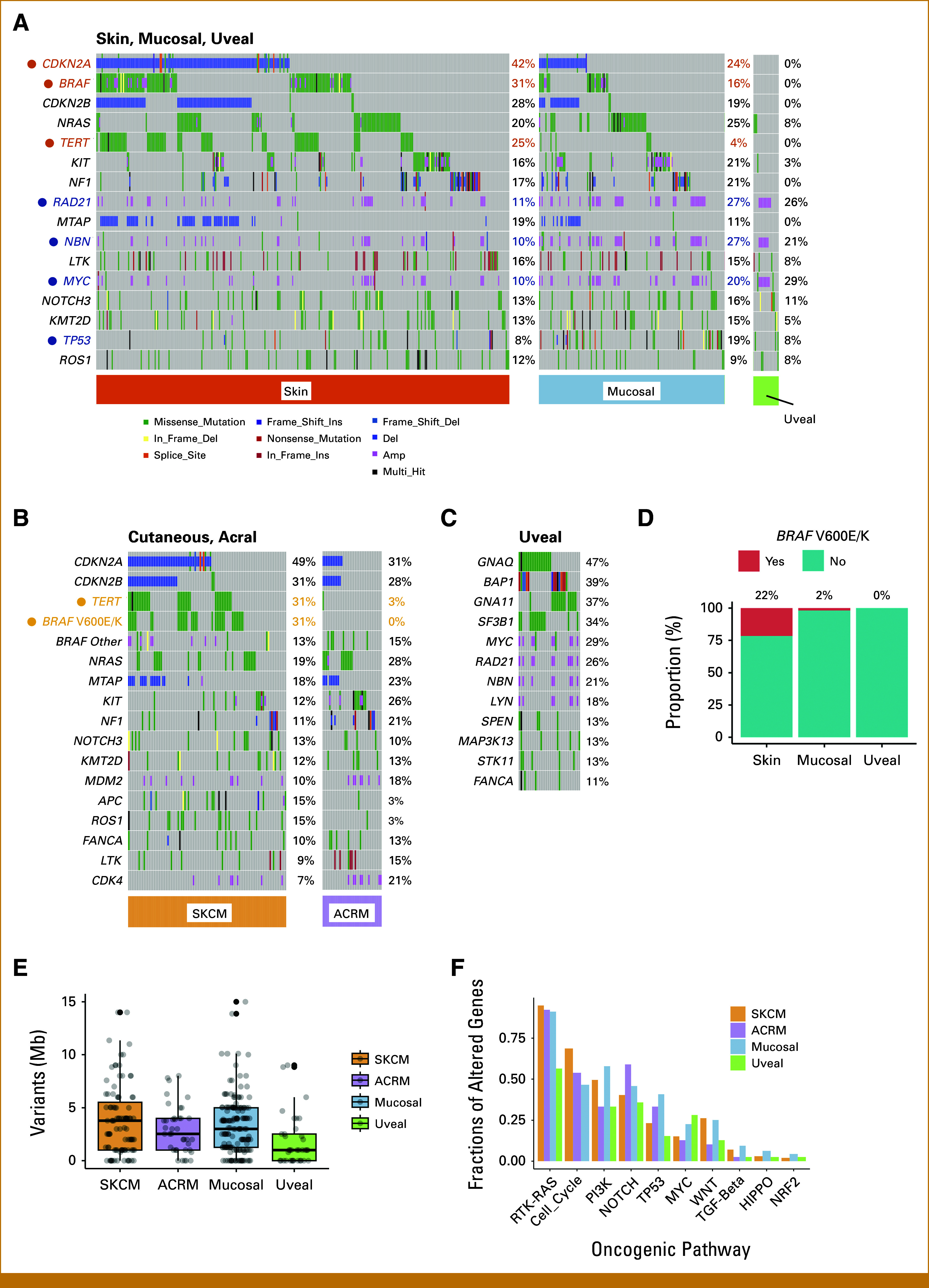
Gene alterations in Japanese patients with skin, mucosal, or uveal melanoma. (A) The most frequently altered genes in all melanomas are shown by tissue subtype. Unique variants of skin and mucosal melanomas are highlighted in orange and blue, respectively. (B) The most frequently altered genes in SKCM and ACRM melanomas are shown by tissue subtype. Unique variants in SKCM are highlighted in orange. (C) The most frequently altered genes in uveal melanomas are shown. (D) Frequency of *BRAF* V600E/K variants in skin, mucosal, and uveal melanomas. (E) The TMB (mean, median/Mb) is shown for SKCM (4.4, 3.8), ACRM (3.0, 2.5), mucosal melanoma (4.4, 3.1), and UM (2.3, 1.0). (F) Fractions of genes altered in oncogenic pathways are shown in SKCM, ACRM, and mucosal and uveal melanomas. ACRM, acral melanoma; SKCM, skin cutaneous melanoma.

**FIG A2.**
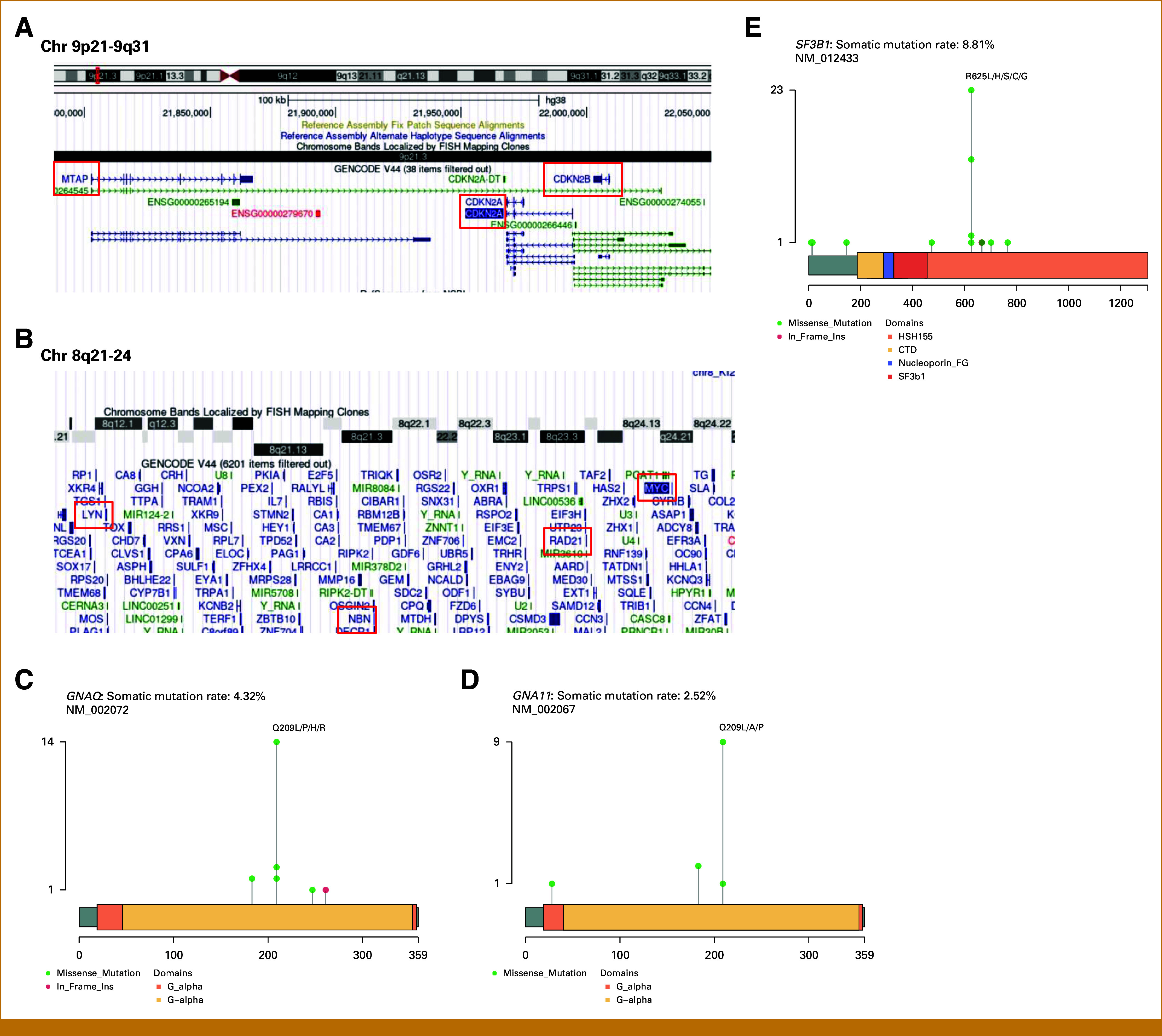
Location of genes in chromosomes and variants in genes. (A) *CDKN2A*, *CDKN2B*, and *MTAP* are located on chromosome 9p21–9q31. (B) *MYC*, *RAD21*, *MBM*, and *LYN* are located on chromosome 8q21–24. (C) Q209 is a hotspot among single-nucleotide variants in *GNAQ.* (D) Q209 is a hotspot among single-nucleotide variants in *GNA11.* (E) R625 is a hotspot among single-nucleotide variants in *SF3B1*.

**FIG A3.**
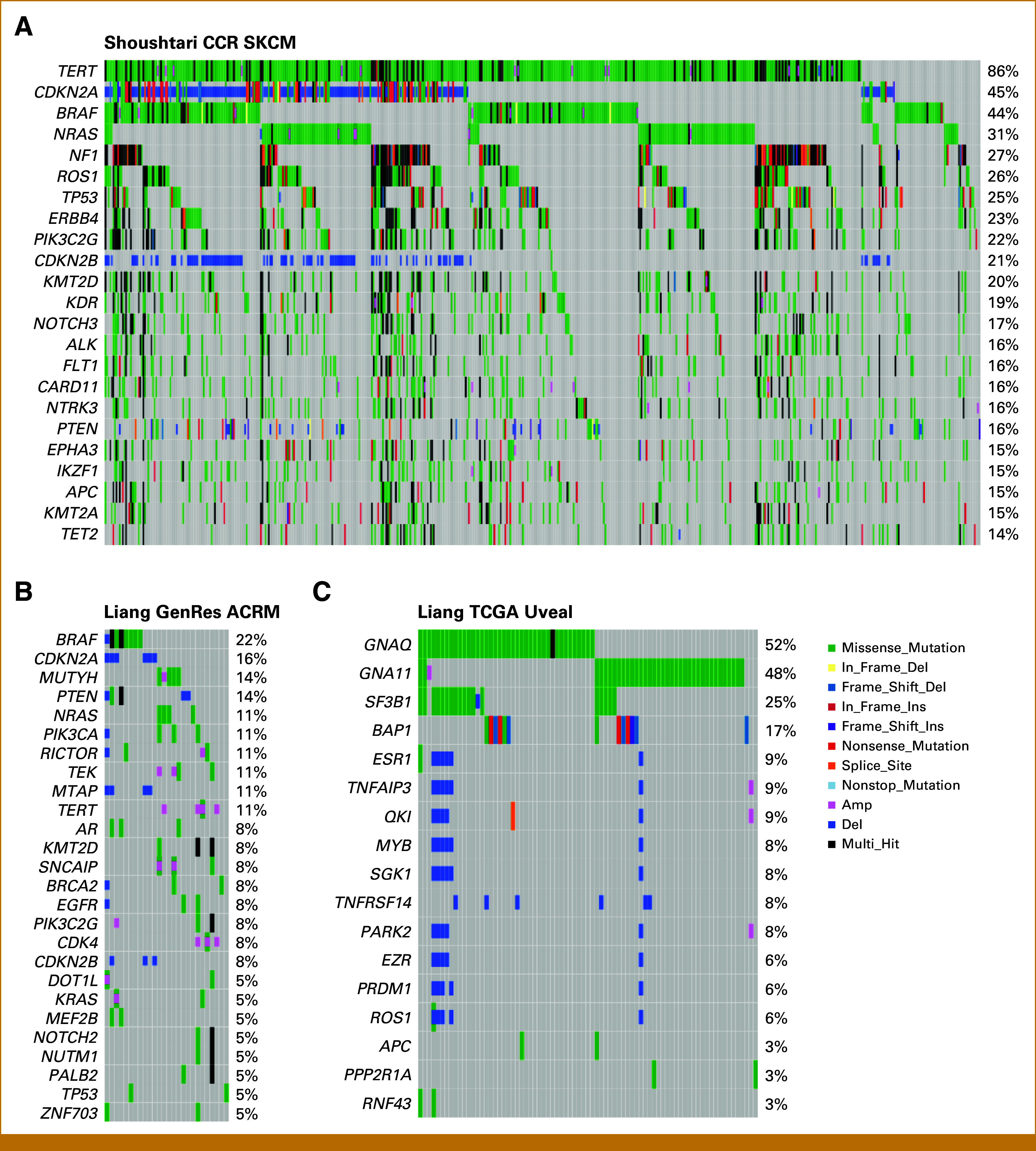
Gene alterations in patients with malignant melanomas in Western countries. (A) Most frequently altered genes in cutaneous melanomas in the literature. (B) Most frequently altered genes in acral melanomas in the literature. (C) Most frequently altered genes in uveal melanomas in the literature.

We began by examining the overall gene alteration landscape of Japanese skin melanoma (n = 364), mucosal melanoma (n = 159), and UM (n = 39; Fig 1A and Appendix Fig A1B). Similar to findings in White melanoma studies, variants in *BRAF* (25%), *NRAS* (20%), *NF1* (17%), and *KIT* (17%) were prevalent in Japanese melanomas (Appendix Fig A1B). Skin melanomas showed a higher frequency of specific gene alterations such as *CDKN2A* deletion (*P* < .05), *BRAF* single-nucleotide variants (SNVs; *P* < .01), and *TERT* promoter variants (*P* < .01) than mucosal melanomas. By contrast, mucosal melanomas predominantly exhibited *TP53* SNVs (*P* < .05) and amplification of *RAD21* (*P* < .01), *NBN* (*P* < .01), and *MYC* (*P* < .05; Fig 1A and Appendix Fig A1C). The exclusivity of variants in *BRAF*, *NRAS*, *NF1*, and *KIT* suggests their roles as driver gene alterations (Fig 1A). The co-occurrence of *CDKN2A* and *CDKN2B* gene deletions, because of their proximity to chromosome 9q21-9q31, and the concurrent amplification of *RAD21*, *NBN*, and *MYC* on chromosome 8q21-24 indicate arm-level gene alterations (Fig 1A, Appendix Figs A2A and A2B). *TERT* promoter variants and *BRAF* V600E/K were found to differentiate between SKCM and ACRM (*P* < .01; Fig 1B). The variant patterns in Japanese SKCM were similar to those in White studies, with *BRAF*, *NRAS*, and *NF1* variants being particularly prevalent in the TCGA SKCM data set, albeit at higher variant rates (Appendix Fig A3A).^24^ The ACRM data suggested higher susceptibility to acquired *KIT* variants in Asians than in White patients (Appendix Fig A3B).^25^ Japanese uveal melanomas exhibited a unique gene alteration landscape, with most cases showing variants in either *GNAQ* or *GNA11*, particularly at Q209 (Fig 1C, Appendix Figs A2C and A2D). These variants were mutually exclusive and were presumed to be driver genes in Japanese uveal melanomas, similar to their roles in White melanomas (Fig 1C and Appendix Fig A3C).^26^ *BAP1* loss-of-function alterations and *SF3B1* SNVs, particularly at R625, were found in over half of the uveal melanomas and may play a supplementary role in oncogenic pathology (Fig 1C and Appendix Fig A2E). Twenty-two percent of skin and 2% of mucosal, but not uveal, melanomas harbored actionable *BRAF* V600E/K variants (Fig 1D). TMB (variants/Mb) across melanoma types was consistently low, with means of 4.42 in SKCM, 2.97 in ACRM, 4.41 in mucosal melanomas, and 1.27 in uveal melanomas (Fig 1E). Among the 10 canonical oncogenic pathways,^27^ variants in the RTK-RAS pathway were the most frequent, followed by those in the cell cycle and PI3K pathways (Fig 1F). Collectively, these data show some genomic similarities and differences between Asian and White melanomas. Notably, Asian and White SKCMs resembled each other in terms of the predominance of *TERT* and *BRAF* V600E/K variants; however, Asian SKCM harbored substantially lower TMB and fewer *BRAF* V600E/K variants.

### Features of *BRAF*, *NRAS*, *NF1*, and *KIT* Variants in Japanese Malignant Melanomas

**FIG 2.**
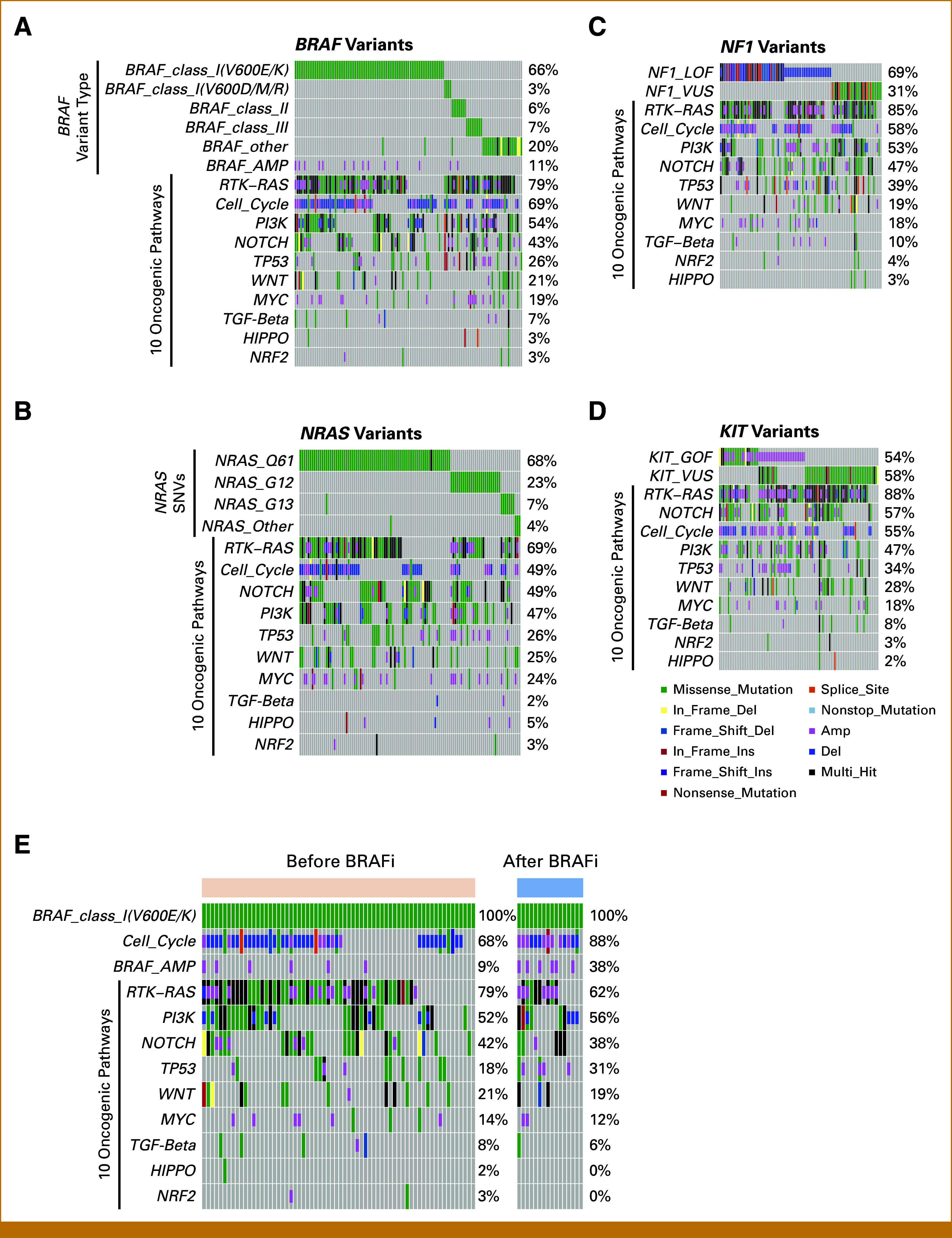
Gene alterations accompanied by driver variants in Japanese melanomas. (A) Among patients with *BRAF* variants, the frequencies of subtypes and other variants in oncogenic pathways are shown. (B) Among patients with *NRAS* variants, the frequencies of subtypes and other variants in oncogenic pathways are shown. (C) Among patients with *NF1* variants, the frequencies of LOF variants, VUS, and other variants in oncogenic pathways are shown. (D) Among patients with *KIT* variants, the frequencies of GOF variants, VUS, and other variants in oncogenic pathways are shown. (E) Among patients with *BRAF* V600E/K variants, the frequency of variants in the cell cycle pathway, *BRAF* amplification, and oncogenic pathway variants in samples collected before and after treatment with BRAF inhibitors are shown. GOF, gain-of-function; LOF, loss-of-function; VUS, variants of unknown significance.

In line with the site-oriented classification, we subsequently investigated gene alteration landscapes using canonical driver genes such as *BRAF*, *NRAS*, *NF1*, and *KIT*. *BRAF* variants are categorized into three classes on the basis of their biologic behavior. Classes I and II are both RAS-independent and consist of active monomers and dimers, respectively.^28^ By contrast, class III variants exhibit minimal or no kinase activity and are RAS-dependent.^29^ The most common *BRAF* variant in our cohort was class I V600E/K, observed in 66% of cases, followed by classes II and III in 6% and 7% of cases, respectively (Fig 2A). *NRAS* was the second most frequently altered gene in our study, with Q61, G12, and G13 variants representing 68%, 23%, and 7% of cases, respectively, and found to be mutually exclusive (Fig 2B). When mutated, the tumor suppressor *NF1* activates the RTK-RAS pathway. Among patients with *NF1* variants, 69% presented with loss-of-function alterations, such as frameshifts, nonsense variants, or gene deletions (Fig 2C). Oncogenic *KIT* variants, as evidenced by OncoKB,^30,31^ were identified in 54% of patients harboring any *KIT*-related alterations, with amplifications accounting for half of these cases (Fig 2D). In summary, among a total of 562 genomic study patients, *NRAS* gain-of-function, *NF1* loss-of-function, and *KIT* gain-of-function alterations were found in 107 (19%), 64 (11%), and 50 (8.9%) patients, respectively. Patients with these variants may benefit from the application of small-molecule inhibitors targeting MEK and c-KIT. In addition, 43 patients (7.7%) harbored oncogenic non-V600E/K *BRAF* variants (ie, classes II and III), which suggests an unmet need for novel drugs targeting these variant classes.

### Gene Alterations in the Specimens From Patients Treated With BRAF Inhibitors

We used clinical data from C-CAT to explore the potential mechanisms of resistance to BRAF inhibitors. Of the patients with *BRAF* V600E/K variants, 16 were treated with BRAF/MEK inhibitors before sample collection and 66 were not. We observed increased frequencies of amplification of cell cycle genes and *BRAF* in specimens from patients previously treated with BRAF/MEK inhibitors (Fig 2E). The prevalence of alterations in the key oncogenic pathways remained consistent before and after treatment (Fig 2E).

### Anticancer Therapies Used in Patients Before MTB Consultation

**FIG 3.**
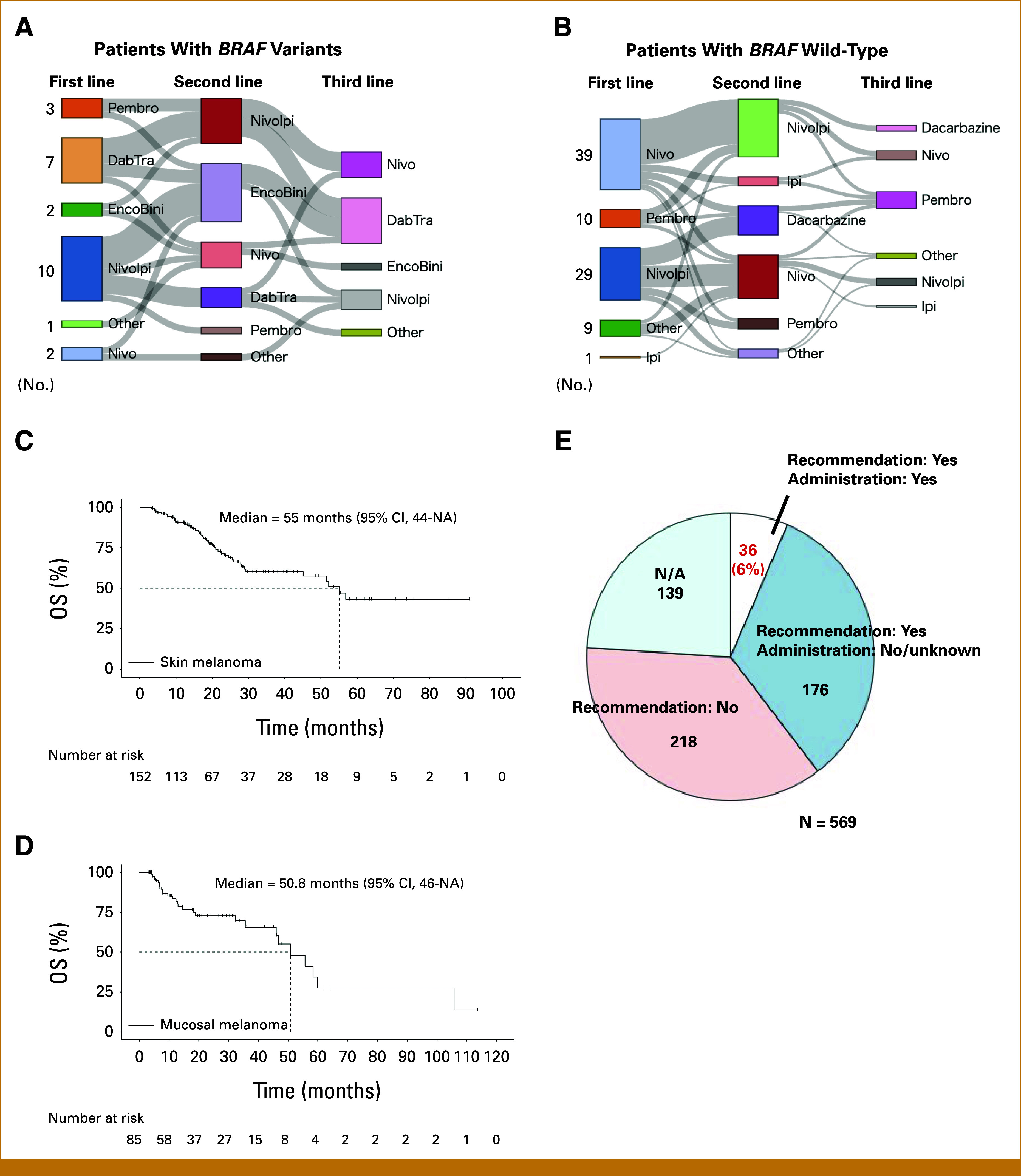
Treatment sequences and real-world outcomes of Japanese patients with malignant melanoma who underwent CGP. (A) Twenty-five patients with advanced *BRAF* V600E/K melanoma received more than one anticancer therapy. (B) Eighty-eight patients with advanced *BRAF* wild-type melanoma received more than one anticancer therapy. (C) Overall survival of patients with advanced skin melanoma is shown. (D) Overall survival of patients with advanced mucosal melanoma is shown. (E) The MTB recommends drugs on the basis of CGP results in some cases. Of 569 patients, 36 (6%) patients received the recommended drugs. NA = Information regarding whether recommendations were provided is not available. CGP, comprehensive genome profiling; DabTra, dabrafenib + trametinib; EncoBini, encorafenib + binimetinib; Ipi, ipilimumab; MTB, molecular tumor board; Nivo, nivolumab; NivoIpi, nivolumab + ipilimumab; Pembro, pembrolizumab.

**FIG A4.**
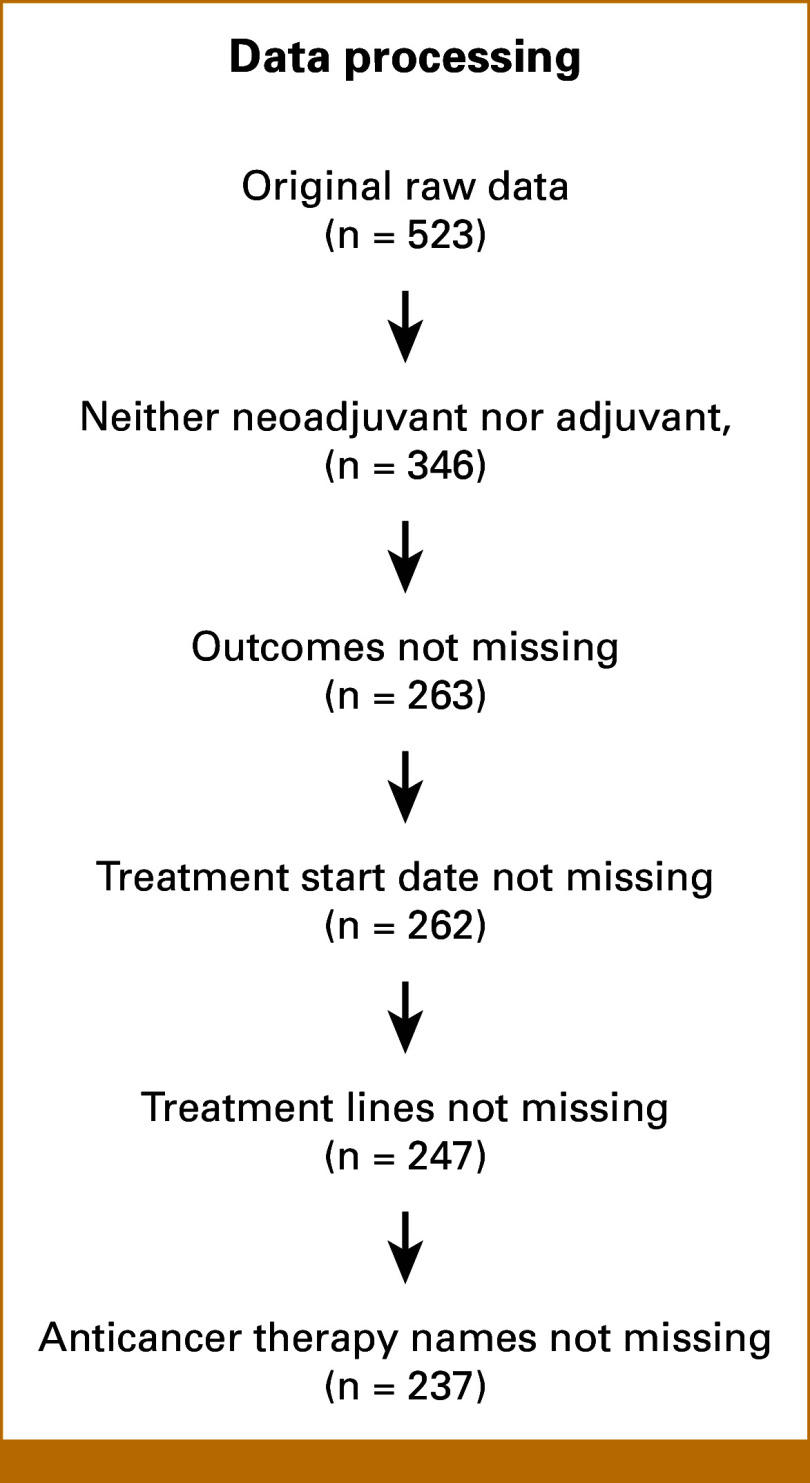
Data filtering methods for clinical assessments. Of the 523 patients, those with a history of neoadjuvant or adjuvant anticancer therapy were excluded. We analyzed cases that retained the outcomes, treatment start dates, treatment lines, and anticancer therapy names.

We reviewed the treatment histories and types of anticancer therapies administered to 237 patients with skin and mucosal melanomas before CGP (Appendix Fig A4). Of the 237 patients, 34 patients had *BRAF* V600E/K variants and 203 did not (Table 2). Of the patients with *BRAF* variants, 44% received first-line BRAF/MEK inhibitors and 50% was treated with first-line ICIs (Table 2). As expected, 94% of those with *BRAF* wild-type received first-line ICIs. CGP was performed before second-line therapy in 26% of the patients with *BRAF* V600E/K and 67% of those with *BRAF* wild-type (*P* < .01; Table 2). A significant proportion of patients with *BRAF* V600E/K variants (47%) received at least three lines of therapy before undergoing CGP, compared with only 13% of those with *BRAF* wild-type (*P* < .001; Table 2). We then investigated anticancer therapy treatment protocols in detail. Among patients with *BRAF* variants, seven of nine patients (78%) treated with first-line BRAF/MEK inhibitors proceeded to second-line ICIs (Fig 3A). Alternatively, 10 of 15 patients (67%) treated with first-line ICIs received BRAF/MEK inhibitors as the second treatment (Fig 3A). Of the patients with *BRAF* wild-type, 55% (16 of 29) and 34% (10 of 29) of patients were treated with second-line anti–PD-1 monotherapy and dacarbazine after the first-line nivolumab + ipilimumab (ICI combination), respectively (Fig 3B). We found that 61% of the patients treated with first-line anti–PD-1 monotherapy received a second-line ICI combination (Fig 3B).

**TABLE 2.** Anticancer Therapies Administered to Patients With or Without *BRAF* V600E/K Before Comprehensive Genome Profiling (top and bottom, respectively)

Anticancer Therapies for Patients With *BRAF* Variants				
Regimen	Lines of Anticancer Therapy, No. (%)			
	1 (n = 34)	2 (n = 25)	3 (n = 16)	4+ (n = 12)
Nivolumab + ipilimumab	11 (32)	7 (28)	2 (13)	1 (8.3)
Nivolumab	3 (8.8)	4 (16)	4 (25)	4 (33)
Pembrolizumab	3 (8.8)	1 (4.0)	0 (0)	0 (0)
Dabrafenib + trametinib	13 (38)	3 (12)	8 (50)	3 (25)
Encorafenib + binimetinib	2 (5.9)	9 (36)	1 (6.3)	3 (25)
Dacarbazine	0 (0)	0 (0)	1 (6.3)	0 (0)
Other	2 (5.9)	1 (4.0)	0 (0)	1 (8.3)

Anticancer Therapies for Patients Without *BRAF* Variants				
Regimen	Lines of Anticancer Therapy, No. (%)			
	1 (n = 203)	2 (n = 88)	3 (n = 26)	4+ (n = 8)
Nivolumab + ipilimumab	81 (40)	32 (36)	4 (15)	0 (0)
Nivolumab	80 (39)	24 (27)	5 (19)	4 (50)
Pembrolizumab	26 (13)	6 (6.8)	9 (35)	0 (0)
Ipilimumab	3 (1.5)	5 (5.7)	1 (3.8)	0 (0)
Dacarbazine	0 (0)	13 (15)	4 (15)	2 (25)
Other	13 (6.4)	8 (9.1)	3 (12)	2 (25)

More patients with skin melanoma were treated with the first-line ICI combination than those with mucosal melanoma (Table 3). This discrepancy may stem from the older average age of the patients with mucosal melanoma compared with the patients with skin melanoma in the study, given the risks associated with the ICI combination. The median survival times of patients with advanced skin and mucosal melanomas in the C-CAT database after data processing (Appendix Fig A4) were 55 months (95% CI, 44 to NA) and 50.8 months (95% CI, 46 to NA), respectively (Figs 3C and 3D).

**TABLE 3.** Characteristics of Patients With Skin and Mucosal Melanomas After Data Processing (Appendix Fig A4)

Characteristic	Skin (n = 152), No. (%)	Mucosal (n = 85), No. (%)
Regimen (first line)		
Nivolumab + ipilimumab	66 (43)	26 (31)
Nivolumab	43 (28)	40 (47)
Pembrolizumab	23 (15)	6 (7.1)
Ipilimumab	1 (0.7)	2 (2.4)
Dabrafenib + trametinib	13 (8.6)	0 (0)
Encorafenib + binimetinib	2 (1.3)	0 (0)
Dacarbazine	0 (0)	0 (0)
Other	4 (2.6)	11 (13)
Event		
Alive	108 (71)	58 (68)
Dead	44 (29)	27 (32)
Age category, years		
<39	11 (7.2)	2 (2.4)
40-59	44 (29)	15 (18)
≥60	97 (64)	68 (80)
*BRAF* V600E/K	34 (22)	0 (0)

### The Role of the MTB in the Treatment of Japanese Melanomas

**FIG A5.**
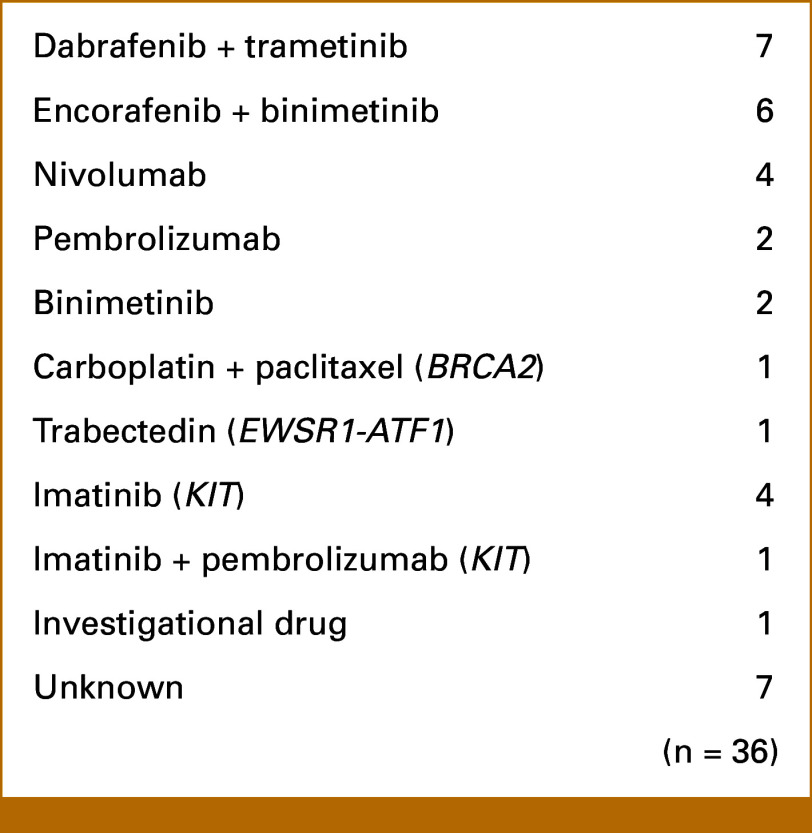
Drugs administered following the molecular tumor board recommendations. Thirty-six patients were administered the recommended drugs. The genes in parentheses are regarded as actionable for the corresponding drugs.

After CGP, the MTB provided recommendations for one third of patients (Fig 3E). However, only six percent of patients were administered the suggested drugs (Fig 3E). Moreover, the MTB discussions predominantly focused on *BRAF* variants, leading to the use of standard BRAF-targeted therapies (Appendix Fig A5). These findings indicate that there is limited patient access to evidence-based drugs and clinical trials, thereby reducing the potential benefits of CGP in Japan.

## DISCUSSION

In this study, we characterized the genomic and clinical features of Asian patients with malignant melanoma on the basis of 569 Japanese cases registered with the C-CAT. Japanese cutaneous melanomas harbored variants in *TERT* promoter regions and *BRAF* in some patients and were associated with sun exposure; however, the frequency was much lower than that in White patients. The TMB was also low, with a median of 3.78/Mb. Arm-level gene alterations in chromosome 8q21-24, where *RAD21*, *NBN*, and *MYC* are located, were found in approximately one fourth of mucosal melanomas. Therefore, gene amplification may abrogate the responses to immunotherapy in patients with mucosal melanoma.^32^ In addition to *GNAQ* and *GNA11*, we found that variants in *SF3B1* were common in Japanese uveal melanomas, which could hamper antitumor T-cell responses in the tumor microenvironment.^33^ Thus, the approval of tebentafusp-tebn in Japan may be beneficial for patients with UM,^34^ and the development of a strategy to overcome such resistance should be explored in the future.

The practical purpose of this study was to identify variants matching clinical trials after treatment with ICIs and BRAF/MEK inhibitors. Indeed, our study revealed that a substantial proportion of patients harbored actionable gene alterations in *NRAS*, *NF1*, and *KIT*. However, most patients did not receive investigational drugs following MTB recommendations. This rate was comparable with that for other types of cancers reported in the United States and Japan,^35,36^ suggesting that a limited number of genomically matched clinical trials are available for melanoma. CGP also identifies the presence of *NTRK* fusions (target of entrectinib^37^ and larotrectinib^38^) and *RET* fusions (target of selpercatinib^39^) although they are rare, with a prevalence of <1%. *NTRK* fusions are relatively common in spitzoid melanomas (approximately 20%).^40^ In fact, one spitzoid melanoma in our study harbored the *LMNA*-*NTRK1* fusion.

As C-CAT stores clinical information and the CGP results, we were able to study real-world trends in anticancer therapies. Interestingly, we found that Japanese physicians chose first-line ICIs in 50% of the patients with *BRAF* V600E/K and first-line BRAF/MEK inhibitors in 44% of them. In general, PD-1 inhibitor with or without CTLA-4 inhibitor is the recommended first-line regimen regardless of *BRAF* variant status.^41,42^ The physicians' decisions might have been influenced by the results of a Japanese retrospective study, suggesting inferior ICI efficacy in Asians with advanced melanoma.^43^ However, it remains to be elucidated in future prospective clinical trials whether immunotherapy or targeted therapy is more suitable for advanced Asian melanoma with *BRAF* V600E/K.

A limitation of this study is the potential selection bias in the C-CAT database. CGP testing is indicated only once for patients with malignant melanoma who (1) have unresectable advanced or recurrent disease and (2) have completed standard anticancer medical treatments (including those expected to complete the treatments). In our study, the frequency of *BRAF* V600E/K was lower than that reported in the Japanese literature.^10^ As shown in Figures 3A and 3B and Table 2, patients with *BRAF* variants received more anticancer therapies than those with *BRAF* wild-type. These results suggest that patients who responded to BRAF/MEK inhibitors well were less likely to undergo CGP. Therefore, although the data are clinically meaningful, the C-CAT database may not represent the entire melanoma landscape in Japan.

In conclusion, our study revealed that actionable gene alterations in *BRAF*, *NRAS*, *KIT*, and *NF1* are common in Japanese patients with melanomas. However, these patients rarely benefited from the CGP in terms of the identification of new medications. These results underscore the importance of improving the accessibility of investigational drugs for clinical trials in Japan.
